# The Toxic Origin of Insanity

**Published:** 1901-11-09

**Authors:** 


### THE TOXIC ORIGIN OF INSANITY

Dr. Ford Robertson[1] again draws attention to the toxic origin of insanity. He describes the primary non-toxic etiological factors in the pathogenesis of insanity as including inherent defects of cerebral organisation; physical causes which directly injure the cerebral neurons, such as traumatism; deficiency of the materials necessary for nerve-cell metabolism; and sensory impulses in some cases; and he maintains that in all other cases, and they form by far the larger proportion, the primary etiolo-

B

gical factor is toxic action. This toxic action may operate either directly upon the nerve cells or indirectly by producing disease of the blood vessels, and thus interfering with nutrition, and it may be caused by exogenous toxic agents, or by infections, or by auto-intoxication and auto-infection. Among exogenous toxic agents we may place alcohol, opium, lead, and many others. The secondary toxæmias which these toxic agents produce are often of great importance. For example, the long-continued use of alcohol may so damage the mucous membrane of the digestive tract as to favour the occurrence of chronic bacterial infection from the gastro-intestinal canal. The secondary toxins so produced, together with the alcohol acting directly, tend to disorder the metabolism in various internal organs, and lead to a condition of poly-intoxication which may even affect the fœtus in utero through the maternal blood. It is probable that this intra-uterine exogenous toxic action establishes the basis of a large group of diseases of the nervous system, including a considerable proportion of cases of idiocy and imbecility, and most cases of idiopathic epilepsy.

The toxic action arising from infection is exemplified in syphilis, influenza, etc., and these again may give rise to secondary toxæmias by way, for example, of gastro-intestinal catarrh, and may also affect the nervous system of the fœtus.

In regard to auto-intoxication, Dr. Robertson wishes to insist upon the paramount importance of that which arises from the gastro-intestinal tract as a cause of insanity. He is strongly inclined to the belief, which he says is supported by a large amount of evidence, that various forms of toxæmia of gastro-intestinal origin are the chief factors in the pathogenesis of a large array of acute and chronic diseases, including several forms of mental disease. These comprise, among others, a large proportion of cases of senile insanity, general paralysis, locomotor ataxy, idiopathic epilepsy (as the determining cause of the fit), and most cases of acute and chronic mania and melancholia. Thus by far the most important factor in the pathogenesis of insanity is toxic action; indeed, the large majority of cases of insanity are not primarily diseases of the brain at all, but are dependent on the action of toxins derived from elsewhere, which affect the functional activity of the cortical nerve-cells by disordering their metabolism and often by damaging them permanently.

[1] Brit. Med. Jour., Oct. 26.

